# Deployment to Military Bases With Open Burn Pits and Respiratory and Cardiovascular Disease

**DOI:** 10.1001/jamanetworkopen.2024.7629

**Published:** 2024-04-25

**Authors:** David A. Savitz, Susan R. Woskie, Anila Bello, Rachel Gaither, Joseph Gasper, Lan Jiang, Christopher Rennix, Gregory A. Wellenius, Amal N. Trivedi

**Affiliations:** 1 Department of Epidemiology, Brown University School of Public Health, Providence, Rhode Island; 2 University of Massachusetts Lowell, Department of Public Health, Lowell; 3 Westat, Rockville, Maryland; 4 Center of Innovation in Long-term Services and Supports for Vulnerable Veterans, Providence VA Medical Center, Providence, Rhode Island; 5 Safety and Occupational Health Applied Sciences Department, Keene State College, Keene, New Hampshire; 6 Alexa Research and Engineering, Washington, District of Columbia; 7 Department of Environmental Health, Boston University School of Public Health, Boston, Massachusetts; 8 Department of Health Services, Policy and Practice, Brown University School of Public Health, Providence, Rhode Island

## Abstract

**Importance:**

Many veterans who served in Afghanistan and Iraq during Operations Enduring Freedom (OEF) and Operation Iraqi Freedom (OIF) were deployed to military bases with open burn pits and exposed to their emissions, with limited understanding of the long-term health consequences.

**Objective:**

To determine the association between deployment to military bases where open burn pits were used for waste disposal and the subsequent risk of developing respiratory and cardiovascular diseases.

**Design, Setting, and Participants:**

This retrospective observational cohort study used Veterans Health Administration medical records and declassified deployment records from the Department of Defense to assess Army and Air Force veterans who were deployed between 2001 and 2011 and subsequently received health care from the Veterans Health Administration, with follow-up through December 2020. Data were analyzed from January 2023 through February 2024.

**Exposure:**

Duration of deployment to military bases with open burn pits.

**Main Outcomes and Measures:**

Diagnosis of asthma, chronic obstructive pulmonary disease, interstitial lung disease, hypertension, myocardial infarction, congestive heart failure, ischemic stroke, and hemorrhagic stroke.

**Results:**

The study population included 459 381 OEF and OIF veterans (mean [SD] age, 31.6 [8.7] years; 399 754 [87.0%] male). Median (IQR) follow-up from end of deployment was 10.9 (9.4-12.7) years. For every 100 days of deployment to bases with burn pits, veterans experienced increased adjusted odds for asthma (adjusted odds ratio [aOR], 1.01; 95% CI, 1.01-1.02), chronic obstructive pulmonary disease (aOR, 1.04; 95% CI, 1.02-1.07), hypertension (aOR, 1.02; 95% CI, 1.02-1.03), and ischemic stroke (aOR, 1.06; 95% CI, 0.97-1.14). Odds of interstitial lung disease, myocardial infarction, congestive heart failure, or hemorrhagic stroke were not increased. Results based on tertiles of duration of burn pit exposures were consistent with those from the continuous exposure measures.

**Conclusions and Relevance:**

In this cohort study, prolonged deployment to military bases with open burn pits was associated with increased risk of developing asthma, COPD, and hypertension. The results also point to a possible increased risk in ischemic stroke. The novel ability to use integrated data on deployment and health outcomes provides a model for additional studies of the health impact of environmental exposures during military service.

## Key Points

**Question:**

Is duration of deployment to military bases with open burn pits associated with an increased risk of diagnosed respiratory and cardiovascular disease?

**Findings:**

In this cohort study of 459 381 military veterans receiving health care from the Veterans Health Administration, deployment to bases with burn pits was associated with modestly increased odds of asthma, chronic obstructive pulmonary disease, hypertension, and ischemic stroke.

**Meaning:**

These findings suggest that prolonged deployment to military bases with open burn pits may increase the risk of developing adverse health outcomes and provide a model for additional studies of the health impact of environmental exposures during military service.

## Teaser

This cohort study assesses the association of deployment to military bases where open burn pits were used for waste disposal and the subsequent risk of developing respiratory and cardiovascular diseases among US veterans receiving care from the Veterans Health Administration.

## Introduction

During Operation Enduring Freedom (OEF) in Afghanistan (2001-2014) and Operation Iraqi Freedom (OIF) in Iraq (2003-2011), the US military used open burn pits on some military bases to dispose of solid, medical, and hazardous materials. In 2009, the Department of Defense (DOD) restricted materials allowed in burn pits, but the US military continued to use burn pits until other methods (eg, incineration, recycling, waste segregation, landfill) were implemented.^1^

Research on the long-term health consequences of exposure to open burn pits has been limited, despite widespread concern among veterans and the general public. While deployment in OEF and OIF has been associated with a higher risk of respiratory disease, exposures other than burn pits could account for this association.^2,3,4^ Isolating the effects of exposure to burn pit emissions is challenging, given the complex mixtures of the emissions that included particulate matter, polycyclic aromatic hydrocarbons, volatile organic compounds, dioxins, and toxic chemicals not typically found in urban air pollution, as well as exposure of military personnel to other airborne hazards, including ambient dust, diesel exhaust, and tobacco use.^5,6^ Comprehensive exposure assessment has not been feasible due to the lack of systematic monitoring or detailed records on burn pit use.

Quantifying the long-term health outcomes associated with burn pit exposure requires a large population of veterans, the ability to isolate any burn pit exposure from other environmental hazards associated with deployment, and individual-level information on burn pit exposure and health outcomes. We identified such an opportunity to reconstruct OEF and OIF veterans’ histories of deployment to bases with and without burn pits and examine long-term health experiences using recently declassified military deployment records from the DOD linked to health outcomes from the Veterans Health Administration (VHA). In this study, we examine the association between deployment to bases with open burn pits and long-term respiratory and cardiovascular disease.

## Methods

This cohort study was reviewed and approved by the Providence VA institutional review board. The research project was found to be exempt from informed consent under Category 4, secondary research, for which consent is not required. This report follows the Strengthening the Reporting of Observational Studies in Epidemiology (STROBE) reporting guideline for cohort studies.

### Study Population

**Figure 1.**
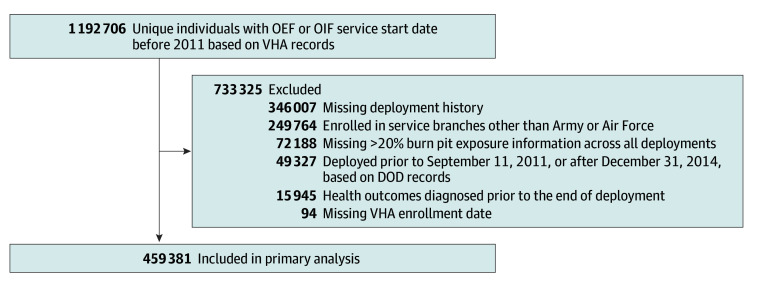
Cohort Enrollment Flowchart DOD indicates Department of Defense; OEF, Operation Enduring Freedom; OIF, Operation Iraqi Freedom; VHA, Veterans Health Administration.

We identified a cohort of Army and Air Force veterans who had first been deployed to OEF or OIF after 2001 and before 2011 and subsequently enrolled in the VHA for health care (Figure 1), excluding Navy and Marine veterans, since needed data were unavailable. The electronic deployment record system was incomplete prior to 2005, so a number of veterans who served only in earlier years were excluded. Starting with VHA administrative and clinical data, we included veterans who had served in OEF or OIF, enrolled in VHA, and had at least 1 VHA health care encounter. We further restricted the cohort to individuals assigned to bases with known exposure status based on DOD records, excluding those who had more than 20% of their cumulative deployment days unassigned due to missing data or incomplete assessment of burn pit utilization. Finally, veterans who were diagnosed with 1 of the conditions of interest before the end of service were excluded to limit the study to incident conditions.

### Deployment History and Assignment of Burn Pit Exposure

As described elsewhere,^1^ DOD deployment records provided information on the specific base names and time periods for an individual’s service. It is the declassification of this information on individual assignment to bases that enabled us to conduct the study. For the 109 most populated bases in Iraq and Afghanistan and 17 outside transit site bases, DOD records were used to determine burn pit use and waste disposal methods for each base from 2001 to 2014, creating a matrix of the waste disposal methods used at each base for each year. Then, for each veteran in each year of deployment, we determined the total days of deployment to a base where burn pits were used and the cumulative days of deployment to bases with burn pits during all OEF and OIF deployments.^1^ We refer to this as *burn pit exposure* for brevity, but in fact, the measure is deployment to bases in which open burn pits were present. Bases where burn pits were not used either shipped waste offsite for disposal or only used onsite incinerators to dispose of waste.

### Health Outcomes

Respiratory and cardiovascular disease diagnoses were determined through VHA health care records between the end of deployment through the end of 2020, using diagnostic algorithms for administrative data identified through literature review of previous validation studies for the diseases of interest (eTable 1 in Supplement 1). Outcomes of interest were the most common forms of respiratory and cardiovascular disease: asthma, chronic obstructive pulmonary disease (COPD), interstitial lung disease, hypertension, myocardial infarction, congestive heart failure, ischemic stroke, and hemorrhagic stroke.

### Covariates

We obtained information on covariates potentially related to respiratory or cardiovascular disease, including demographic factors (age at VHA enrollment, sex, race and ethnicity, US Census region), obesity (defined as body mass index [BMI; calculated as weight in kilograms divided by height in meters squared] ≥30) when VHA care was first received, cigarette smoking, priority category for VHA eligibility, branch of service, year of initial VHA care, total days of deployment, and the median household income and percentage with college education in their Census tract at the time of initial VHA health care (eTable 7 in Supplement 1). Race and ethnicity were obtained from the Corporate Data Warehouse and categorized as Black, Hispanic, White, and other (eg, American Indian or Alaska native, Asian, Native Hawaiian or Other Pacific Islander). Black race and Hispanic ethnicity were included as identifiers of socially disadvantaged groups in the US. Cigarette smoking (categorized each year of care as yes or no and summarized across years as never, always, or mixed), VHA priority status for receiving care, branch of service (Army or Air Force), and rank (enlisted, officer, unknown) were derived from VHA records. Total duration of deployment was derived from DOD records. Because health problems may reduce the probability of continued deployment, individuals who were deployed for longer periods had more favorable subsequent health experiences than those deployed for shorter periods (called the *healthy deployer effect*). Since the total duration of deployment was strongly correlated with duration of deployment to bases with burn pits (correlation coefficient, 0.71), we adjusted for duration of deployment and duration of deployment squared and cubed to control for linear and nonlinear effects.

### Statistical Analysis

We used multivariable logistic regression, with and without adjustment for covariates, to determine the association between deployment to bases with burn pits and the odds of being diagnosed with diseases of interest. Our approach for interpreting data is based on an evaluation of the magnitude, direction, and precision of the effect estimates, rather than binary significance testing.^7^ Exposure was defined as the total number of days deployed to bases that used burn pits for waste disposal during the period the individual was assigned to that base. Exposure was assessed both continuously (expressed in units of 100 days of exposure) and categorically (no deployment to bases with burn pits) and tertiles of nonzero days burn pit exposure duration. Disease outcomes were dichotomized as ever or never diagnosed.

Several sensitivity analyses were conducted to address specific methodological concerns. We examined characteristics of individuals who were excluded from analysis for various reasons, mostly due to missing deployment history or service outside the Army or Air Force (eTable 3 in Supplement 1), and individuals who had indications of receiving VHA care prior to the end of deployment (eTable 4 in Supplement 1). To address concerns with potential health-based decisions regarding deployment, we conducted analyses of exposure and disease after excluding veterans who were never deployed to Iraq or Afghanistan, those who enrolled for VHA care prior to the end of deployment, and those who did not have a mean of at least 1 health care encounter per year (eTable 5 in Supplement 1). To examine sources of confounding, we conducted analyses that adjusted only for total duration of deployment and only for covariates other than total duration of deployment separately (eTable 6 in Supplement 1).

Analyses were conducted using SAS Enterprise Guide version 8.3 (SAS Institute). Data were analyzed from January 2023 through February 2024.

## Results

A total of 459 381 individuals, and all available service records between September 11, 2001, and December 31, 2014, were assessed, with follow-up data from the VHA through December 31, 2020 (Figure 1). The study population had a mean (SD) age of 31.6 (8.7) years (median [IQR] age, 29 [25-38] years) at entry into VHA care and was predominantly male (399 754 [87.0%] male). The study population was racially and ethnically diverse, including 75 258 Black veterans (16.4%), 52 527 Hispanic veterans (11.4%), and 305 996 White veterans (66.6%). There was substantial variability in socioeconomic status indicators (Table 1; eTable 2 in Supplement 1). More than one-third of veterans had BMI greater than 30 (155 770 veterans [33.9%]), and nearly half had smoked cigarettes at least some of the time (199 229 veterans [43.4%]). Most cohort members had served in the Army (433 065 veterans [94.3%]). Except for indicators of military service, there were only modest differences between individuals with no exposure to bases with burn pits vs those in the low or middle exposure or high exposure duration groups (Table 1).

**Table 1.** Cohort Sociodemographic and Military Service Characteristics Stratified by Burn Pit Exposure

Characteristic	Individuals, No. (%)			
	Overall (N = 459 381)	Burn pit exposure^a^		
		None (n = 66 516)	Lowest and middle tertiles (n = 262 097)	Highest tertile (n = 130 768)
Age at VHA enrollment, mean (SD)	31.6 (8.7)	32.8 (9.6)	31.2 (8.8)	32.0 (8.1)
Sex				
Male	399 754 (87.0)	55 471 (83.4)	227 335 (86.7)	116 948 (89.4)
Female	57 074 (12.4)	10 694 (16.1)	33 229 (12.7)	13 151 (10.1)
Missing	2553 (0.6)	351 (0.5)	1533 (0.6)	669 (0.5)
Race and ethnicity				
Black	75 258 (16.4)	11 685 (17.6)	40 653 (15.5)	22 920 (17.5)
Hispanic	52 527 (11.4)	7563 (11.4)	29 550 (11.3)	15 414 (11.8)
White	305 996 (66.6)	43 015 (64.7)	177 731 (67.8)	85 250 (65.2)
Other^b^	540 (0.1)	84 (0.1)	296 (0.1)	160 (0.1)
Missing	25 060 (5.5)	4169 (6.3)	13 867 (5.3)	7024 (5.4)
BMI				
<25	90 971 (19.8)	13 284 (20.0)	54 534 (20.8)	23 153 (17.7)
25-30	165 162 (36.0)	24 286 (36.5)	95 062 (36.3)	45 814 (35.0)
>30	155 770 (33.9)	21 066 (31.7)	85 009 (32.4)	49 695 (38.0)
Missing	47 478 (10.3)	7880 (11.8)	27 492 (10.5)	12 106 (9.3)
Cigarette smoking				
Never	186 351 (40.6)	29 127 (43.8)	104 836 (40.0)	52 388 (40.1)
Sporadic	93 631 (20.4)	12 575 (18.9)	54 445 (20.8)	26 611 (20.3)
Always	105 598 (23.0)	12 964 (19.5)	61 226 (23.4)	31 408 (24.0)
Missing	73 801 (16.1)	11 850 (17.8)	41 590 (15.9)	20 361 (15.6)
Branch of service				
Air Force	26 316 (5.7)	4330 (6.5)	13 305 (5.1)	8681 (6.6)
Army	433 065 (94.3)	62 186 (93.5)	248 792 (94.9)	122 087 (93.4)
Rank				
Enlisted	408 571 (88.9)	56 787 (85.4)	231 994 (88.5)	119 790 (91.6)
Officer or warrant officer	37 161 (8.1)	7138 (10.7)	20 950 (8.0)	9073 (6.9)
Unknown	13 649 (3.0)	2591 (3.9)	9153 (3.5)	1905 (1.5)
Deployment location				
Afghanistan only	72 448 (15.8)	3447 (5.2)	54 200 (20.7)	14 801 (11.3)
Iraq only	274 810 (59.8)	26 228 (39.4)	179 721 (68.6)	68 861 (52.7)
Both Afghanistan and Iraq	73 628 (16.0)	951 (1.4)	26 063 (9.9)	46 614 (35.6)
Neither Afghanistan nor Iraq	38 495 (8.4)	35 890 (54.0)	2113 (0.8)	492 (0.4)
Total deployment duration, median (IQR), d	353 (277-569)	290 (207-357)	327 (259-394)	651 (455-793)
Calendar year of VHA enrollment				
Before 2007	69 213 (15.1)	13 542 (20.4)	42 055 (16.0)	13 616 (10.4)
2007-2009	107 779 (23.5)	17 151 (25.8)	65 359 (24.9)	25 269 (19.3)
2010-2012	134 095 (29.2)	18 424 (27.7)	79 926 (30.5)	35 745 (27.3)
After 2012	148 294 (32.3)	17 399 (26.2)	74 757 (28.5)	56 138 (42.9)

Abbreviations: BMI, body mass index (calculated as weight in kilograms divided by height in meters squared); VHA, Veterans Health Administration.

^a^ Tertiles were defined as lowest, 1 to 213 days; middle, 214 to 339 days; and highest, 340 to 3178 days.

^b^ Includes American Indian or Alaska native, Asian, Native Hawaiian or Other Pacific Islander.

Most of the cohort had been assigned to bases with burn pits at some time (392 865 veterans [85.6%]; overall median [IQR] exposure, 244 [98-362] days), and the median (IQR) duration of deployment to bases with burn pits within the tertiles was 129 (66-181) days in the lowest exposure group, 272 (245-308) days in the middle exposure group, and 474 (395-618) days in the highest exposure group. More than half of those with no documented exposure to burn pits were deployed to countries other than Afghanistan or Iraq (eg, transit bases mostly in the region) (35 890 veterans [54.0%]). The median (IQR) duration of deployment was 353 (277-569) days for the full cohort, with medians of 290 (207-357) days for the no exposure group, 281 (205-368) for the lowest exposure group, 342 (293-419) days for the middle exposure group, and 651 (455-793) days for the highest exposure group. Median (IQR) follow-up from end of deployment was 10.9 (9.4-12.7) years.

**Figure 2.**
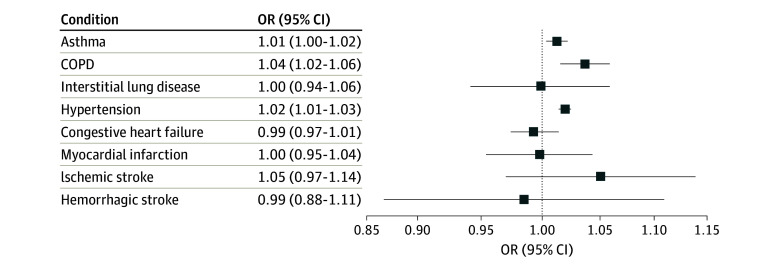
Associations of Open Burn Pit Exposure With Respiratory and Cardiovascular Disease Outcomes in a Cohort of US Military Veterans (N = 459 381) All odds ratios (ORs) are adjusted for age at Veterans Health Administration enrollment, sex (male or female), race and ethnicity (Black or not Black), region (Northeast, Midwest, South, West), median income of Census tract, percentage with a bachelor’s degree in Census tract, body mass index (calculated as weight in kilograms divided by height in meters squared; >30 or ≤30), cigarette smoking (never, always, or mixed), Veterans Health Administration enrollment priority (service connected or disability, low income, low priority or high copay), branch of service (Army or Air Force), rank (officer, enlisted, or unknown), year of entry for Veterans Health Administration care, deployment duration, square of deployment duration, and cube of deployment duration.

The associations between duration of time deployed to bases where burn pits were in use and health outcomes are summarized in Figure 2, Table 2, and Table 3. Prior to adjustment, all outcomes except asthma were inversely associated with duration of burn pit exposure; however, in fully adjusted analyses, each 100 days of burn pit exposure was associated with small increases in the odds of having asthma (adjusted odds ratio [aOR], 1.01; 95% CI, 1.00-1.02) and COPD (aOR, 1.04; 95% CI, 1.02-1.06) but not interstitial lung disease (Figure 2). The patterns across tertiles were consistent with the results based on the continuous exposure measure, with modest dose-response gradients (Table 2).

**Table 2.** Odds of Respiratory Disease by Duration of Burn Pit Exposure

Measure	Diagnoses, No. (N = 459 381)	Unadjusted risk, %	Unadjusted OR (95% CI)	Adjusted OR (95%CI)^a^
**Asthma**				
Overall	23 269	5.07	NA	NA
By tertile of exposure^b^				
None	3333	5.01	1 [Reference]	1 [Reference]
Lowest	6678	5.10	1.02 (0.98-1.06)	1.05 (1.01-1.10)
Middle	6679	5.09	1.02 (0.98-1.06)	1.05 (1.01-1.10)
Highest	6579	5.03	1.00 (0.96-1.05)	1.10 (1.04-1.16)
**Chronic obstructive pulmonary disease**				
Overall	4237	0.92	NA	NA
By tertile of exposure^b^				
None	675	1.01	1 [Reference]	1 [Reference]
Lowest	1330	1.02	1.00 (0.91-1.10)	1.16 (1.05-1.27)
Middle	1248	0.95	0.94 (0.85-1.03)	1.18 (1.07-1.30)
Highest	984	0.75	0.74 (0.67-0.82)	1.22 (1.08-1.38)
**Interstitial lung disease**				
Overall	461	0.10	NA	NA
By tertile of exposure^b^				
None	78	0.12	1 [Reference]	1 [Reference]
Lowest	146	0.11	0.95 (0.72-1.25)	1.11 (0.84-1.46)
Middle	137	0.10	0.89 (0.67-1.18)	1.11 (0.83-1.49)
Highest	100	0.08	0.65 (0.49-0.88)	0.88 (0.62-1.26)

Abbreviations: NA, not applicable; OR, odds ratio.

^a^ Adjusted for age at Veterans Health Administration enrollment, sex (male or female), race and ethnicity (Black or not Black), region (Northeast, Midwest, South, West), median income of Census tract, percentage with a bachelor’s degree in Census tract, body mass index (calculated as weight in kilograms divided by height in meters squared; >30 or ≤30), cigarette smoking (never, always, or mixed), Veterans Health Administration enrollment priority (service connected or disability, low income, low priority or high copay), branch of service (Army or Air Force), rank (officer, enlisted, or unknown), year of entry for Veterans Health Administration care, deployment duration, square of deployment duration, and cube of deployment duration.

^b^ Tertiles were defined as lowest, 1 to 213 days (n = 130 933); middle, 214 to 339 days (n = 131 164); and highest, 340 to 3178 days (n = 130 768). There were 66 516 veterans with no exposure.

**Table 3.** Odds of Cardiovascular Disease by Duration of Burn Pit Exposure

Measure	Diagnoses, No. (n = 459 381)	Unadjusted risk, %	Unadjusted OR	Adjusted OR (95% CI)^a^
**Hypertension**					
Overall	73 337	15.96	NA	NA
By tertile of exposure^b^				
None	11 569	17.39	1 [Reference]	1 [Reference]
Lowest	20 800	15.89	0.90 (0.88-0.92)	1.00 (0.97-1.03)
Middle	20 450	15.59	0.88 (0.86-0.90)	1.05 (1.02-1.08)
Highest	20 518	15.69	0.88 (0.86-0.91)	1.10 (1.07-1.14)
**Congestive heart failure**					
Overall	4152	0.90	NA	NA
By tertile of exposure^b^				
None	731	1.10	1 [Reference]	1 [Reference]
Lowest	1255	0.96	0.87 (0.79-0.95)	0.97 (0.88-1.07)
Middle	1179	0.90	0.82 (0.74-0.90)	0.99 (0.89-1.09)
Highest	987	0.75	0.68 (0.62-0.75)	0.94 (0.83-1.06)
**Myocardial infarction**					
Overall	955	0.21	NA	NA
By tertile of exposure^b^				
None	174	0.26	1 [Reference]	1 [Reference]
Lowest	315	0.24	0.92 (0.76-1.11)	1.02 (0.85-1.23)
Middle	270	0.21	0.79 (0.65-0.95)	0.96 (0.78-1.17)
Highest	196	0.15	0.57 (0.47-0.70)	0.93 (0.72-1.19)
**Ischemic stroke**					
Overall	328	0.07	NA	NA
By tertile of exposure^b^				
None	48	0.07	1 [Reference]	1 [Reference]
Lowest	106	0.08	1.12 (0.80-1.58)	1.28 (0.91-1.80)
Middle	103	0.08	1.09 (0.77-1.53)	1.4 (0.97-2.00)
Highest	71	0.05	0.75 (0.52-1.09)	1.44 (0.93-2.25)
**Hemorrhagic stroke**					
Overall	116	0.03	NA	NA
By tertile of exposure^b^				
None (n = )	19	0.03	1 [Reference]	1 [Reference]
Lowest	34	0.03	0.91 (0.52-1.59)	0.97 (0.55-1.71)
Middle	35	0.03	0.93 (0.53-1.63)	1.08 (0.60-1.96)
Highest	28	0.02	0.75 (0.42-1.34)	0.97 (0.47-1.99)

Abbreviations: NA, not appliable; OR, odds ratio.

^a^ Adjusted for age at Veterans Health Administration enrollment, sex (male or female), race and ethnicity (Black or not Black), region (Northeast, Midwest, South, West), median income of Census tract, percentage with a bachelor’s degree in Census tract, body mass index (calculated as weight in kilograms divided by height in meters squared; >30 or ≤30), cigarette smoking (never, always, or mixed), Veterans Health Administration enrollment priority (service connected or disability, low income, low priority or high copay), branch of service (Army or Air Force), rank (officer, enlisted, or unknown), year of entry for Veterans Health Administration care, deployment duration, square of deployment duration, and cube of deployment duration.

^b^ Tertiles were defined as lowest, 1 to 213 days (n = 130 933); middle, 214 to 339 days (n = 131 164); and highest, 340 to 3178 days (n = 130 768). There were 66 516 veterans with no exposure.

Hypertension also was associated with duration of burn pit exposure based on a continuous (aOR per 100 days of exposure, 1.02; 95% CI, 1.01-1.03) (Figure 2) and categorical exposure measure (middle exposure group: aOR, 1.05; 95% CI, 1.02-1.08; highest exposure group: aOR, 1.10; 95% CI, 1.07-1.14) (Table 3). An elevated risk of ischemic stroke was observed (Figure 2, Table 3), but with limited precision. There were no noteworthy associations between burn pit exposure and other forms of cardiovascular disease.

Individuals who were excluded from the cohort (eTable 3 in Supplement 1) or those who had indications of VHA care prior to the end of deployment (eTable 4 in Supplement 1) were similar to the rest of the cohort on sociodemographic characteristics. Exclusion of individuals who were never deployed to Afghanistan or Iraq, who enrolled for VHA care prior to discharge, or who had a mean of less than 1 health care encounter per year had little impact on the measures of associations between deployment to bases with burn pits and disease outcomes (eTable 5 in Supplement 1). Adjustment for duration of deployment largely accounted for the differences between unadjusted and adjusted associations for the health outcomes with a positive association, with the exception of hypertension, which was only increased with adjustment for all the covariates (eTable 6 in Supplement 1).

## Discussion

Results from this cohort study indicate that longer deployment to military bases with open burn pits was associated with small increases in risk of asthma, COPD, and hypertension among veterans who served in Iraq and Afghanistan during OEF and OIF. While the associations were modest in magnitude, there are several million potentially affected veterans, making any elevation in risk important to document.

Three previous studies have attempted to directly examine the association between burn pit exposures and health outcomes.^8,9,10^ The Millennium Cohort study used survey data to assess the association between deployment to 2 large bases that had used burn pits compared with deployment to other locations that did not.^8^ They did not find an association between burn pit exposure and self-reported respiratory symptoms or diagnoses but only had a mean follow-up time of 2.9 years between discharge, compared with a median of 10.9 years in our study. Using the Airborne Hazards and Open Burn Pit Registry, Liu et al^9^ identified several positive associations, but the registry is of limited value due to the highly self-selected population. Rohrbeck et al^10^ conducted a study of 200 active-duty service members and did not identify associations between deployment to bases with burn pits and respiratory disease outcomes, but the follow-up only continued during time in service and the comparison group consisted of individuals not deployed at all rather than those deployed to bases without burn pits.

Our study provides important new information on the long-term health outcomes associated with burn pit exposures due to strengths that distinguish it from previous analyses: a much larger population, individual-level reconstruction of each veteran’s history of deployment to bases with burn pits using declassified military records, and a long follow-up time for assessment of health outcomes. The use of the VHA health care records identified clinical diagnoses over the entire period after initial enrollment, minimizing loss to follow-up or dependence on self-reported health conditions. Our goal was to assess a potential direct association of deployment to bases with burn pits on future health outcomes, isolated from any biases that would create a spurious association. Only if the decision to assign individuals to bases with vs without burn pits were independently associated with future health outcomes, which seems unlikely, would the association between burn pit exposure and disease be subject to confounding. A series of sensitivity analyses addressed concerns with potential selective deployment or other potential biases related to utilization of VHA health care and the results were consistent with the original findings.

### Limitations

This study has several limitations. First, the positive associations we identified were sensitive to adjustment for total duration of deployment. The combination of a strong association between duration of burn pit exposure and total duration of deployment and a reduced risk of disease among individuals with a longer period of deployment (healthy deployer effect) appears to introduce confounding for all the outcomes, which was the rationale for relying on the fully adjusted results. Second, we lacked data on burn pit characteristics at each base (eg, volume, frequency, content) that could impact the probability of exposure to specific toxicants among individuals serving at the base. Nor did we have data to adjust each individual’s likely exposure intensity based on their base location or job tasks. Therefore, we assumed a constant exposure intensity within the year and equal exposures for each individual assigned a burn pit base, due to the lack of data on duration or volume of burning, individual distance from the burn pit, prevailing weather conditions, or job duties that impacted the likelihood and intensity of potential burn pit exposures. While the presence of a burn pit was assigned for a full calendar year for each base, the burn pit may have been operational for only part of the year. Resulting exposure inaccuracy is unlikely to vary in relation to health status and generally (but not always); this nondifferential exposure misclassification would likely bias results toward the null. Third, although we assumed that assignment to bases with or without burn pits would be unrelated to health status at the time of assignment, we do not have direct information to confirm this assumption. To the extent that assignment to bases with or without burn pits is independently associated with future health status, the results would be biased.

Fourth, we were only able to include veterans who obtained health care and had a diagnosis made through the VHA, estimated at 60% of OEF and OIF veterans.^11^ There is little reason to believe that the association between burn pit exposure and health outcomes would differ in the VHA subpopulation relative to the total population deployed. We were unable to identify conditions that were exclusively treated outside the VHA, subclinical indicators of health impairment (eg, pulmonary function), or conditions not medically identified. Given the serious nature of the health outcomes of concern (eg, COPD, myocardial infarction) or persistence of those that are sometimes less severe (eg, asthma, hypertension), it is likely that even veterans who obtained some of their health care outside the VHA would have been identified in the VHA records as having developed the diseases of interest. The diagnostic algorithms that we used had been developed and tested previously with positive predictive values between 60% and 90%, most likely to be nondifferential with respect to history of deployment to bases with burn pits but with a potential for having that history influence diagnosis. Specific health conditions that have been reported to be associated with burn pit exposure, such as constrictive bronchiolitis, are rare and require detailed clinical data that were unavailable in our study.^12^ We chose to focus on more common but less specific categories of respiratory and pulmonary disease.

Additionally, it should be noted that we lacked the data needed to conduct a longitudinal analysis from the time of deployment through the end of the follow-up period or death. We did not have information on health conditions arising before or during deployment, nor were we able to follow veterans prospectively from discharge to the onset of health care within the VHA. Thus, the time of first diagnosis was uncertain and instead we focused on whether a condition was ever diagnosed. While this precludes considering timing of exposure in relation to disease onset, it is not likely that the gaps in knowledge would apply differently to those assigned to bases with or without burn pits.

## Conclusions

The findings of this cohort study suggest that health care for veterans of OEF and OIF should consider the potential health outcomes associated with exposure to emissions from open burn pits, with implications for access to care and benefits. Potential long-term health outcomes associated with open burn pit exposure call for continued efforts to evaluate whether there are measurable increases in disease risk. Our ability to use recently declassified DOD deployment histories that provide base assignments for studying veterans’ health is an important milestone in conducting research on health outcomes associated with exposures occurring during military service. The DOD has invested considerable effort into facilitating the use of deployment data for environmental health research, and this novel resource lends itself to addressing other potential health consequences of deployment-related exposures.

## Notes

Supplement 1. **eTable 1.** Algorithms for Disease Diagnoses **eTable 2.** Additional Cohort Sociodemographic and Military Service Characteristics in Relation to Burn Pit Exposure **eTable 3.** Comparison of Sociodemographic and Military Service Characteristics Between Individuals Included in the Analytic Sample and Those Excluded Due to Missing Data for Whom Characteristics Were Available **eTable 4.** Characteristics of Cohort Members Who Received VHA Care Prior to End of Deployment Compared With Those Who Only Received Care After the End of Deployment, Including Breakdown by Priority Status **eTable 5.** Adjusted Odds Ratios for Respiratory and Cardiovascular Disease by Duration of Burn Pit Exposure, Excluding Those Never Deployed to Iraq or Afghanistan, Excluding Those Enrolled for VHA Care Prior to the End of Deployment, Restricted to Those Who Had a Mean of More Than 1 Health Care Encounter per Year of Follow-Up, or Restricted to Those With No Missing Deployment History Data **eTable 6.** Odds Ratios for Respiratory and Cardiovascular Disease by Duration of Burn Pit Exposure, Adjusted for all Covariates, Duration of Deployment Only, or for Nonduration Covariates Only **eTable 7.** Sources of Covariate Data and Timing of Variable Ascertainment
eReferences.

Supplement 2.
Data Sharing Statement
