# Supplementary material for: Deployment to Military Bases With Open Burn Pits and Respiratory and Cardiovascular Disease
Source: JAMA Netw Open. 2024 Apr 25;7(4):e247629. doi: 10.1001/jamanetworkopen.2024.7629 (PMC11046344; doi:10.1001/jamanetworkopen.2024.7629)
Supplement: Supplement 1. — eTable 1. Algorithms for Disease Diagnoses eTable 2. Additional Cohort Sociodemographic and Military Service Characteristics in Relation to Burn Pit Exposure eTable 3. Comparison of Sociodemographic and Military Service Characteristics Between Individuals Included in the Analytic Sample and Those Excluded Due to Missing Data for Whom Characteristics Were Available eTable 4. Characteristics of Cohort Members Who Received VHA Care Prior to End of Deployment Compared With Those Who Only Received Care After the End of Deployment, Including Breakdown by Priority Status eTable 5. Adjusted Odds Ratios for Respiratory and Cardiovascular Disease by Duration of Burn Pit Exposure, Excluding Those Never Deployed to Iraq or Afghanistan, Excluding Those Enrolled for VHA Care Prior to the End of Deployment, Restricted to Those Who Had a Mean of More Than 1 Health Care Encounter per Year of Follow-Up, or Restricted to Those With No Missing Deployment History Data eTable 6. Odds Ratios for Respiratory and Cardiovascular Disease by Duration of Burn Pit Exposure, Adjusted for all Covariates, Duration of Deployment Only, or for Nonduration Covariates Only eTable 7. Sources of Covariate Data and Timing of Variable Ascertainment eReferences. [file jamanetwopen-e247629-s001.pdf]

Supplemental Online Content

Savitz DA, Woskie SR, Bello A, et al. Deployment to military bases with open burn pits and respiratory and cardiovascular disease. *JAMA Netw Open*. 2024;7(4):e247629. doi:10.1001/jamanetworkopen.2024.7629

eTable 1. Algorithms for Disease Diagnoses

eTable 2. Additional Cohort Sociodemographic and Military Service Characteristics in Relation to Burn Pit Exposure

eTable 3. Comparison of Sociodemographic and Military Service Characteristics Between Individuals Included in the Analytic Sample and Those Excluded Due to Missing Data for Whom Characteristics Were Available

eTable 4. Characteristics of Cohort Members Who Received VHA Care Prior to End of Deployment Compared With Those Who Only Received Care After the End of Deployment, Including Breakdown by Priority Status

eTable 5. Adjusted Odds Ratios for Respiratory and Cardiovascular Disease by Duration of Burn Pit Exposure, Excluding Those Never Deployed to Iraq or Afghanistan, Excluding Those Enrolled for VHA Care Prior to the End of Deployment, Restricted to Those Who Had a Mean of More Than 1 Health Care Encounter per Year of Follow-Up, or Restricted to Those With No Missing Deployment History Data

eTable 6. Odds Ratios for Respiratory and Cardiovascular Disease by Duration of Burn Pit Exposure, Adjusted for all Covariates, Duration of Deployment Only, or for Nonduration Covariates Only

eTable 7. Sources of Covariate Data and Timing of Variable Ascertainment

eReferences.

This supplemental material has been provided by the authors to give readers additional information about their work.

© 2024 Savitz DA et al. *JAMA Network Open.*

Table S1. Algorithms for disease diagnoses

Condition	Algorithm	ICD-9 Diagnostic Codes	ICD-10 Diagnostic Codes	Accuracy	Sources
Asthma	2 outpatient ICD codes or 1 inpatient ICD code	493.0x, 493.1x, 493.9x	J45.x	PPV: 60.4%<br>Sensitivity: 85%<br>Specificity: 75.1%	Dombkowski et al. 2009[1], Gershon et al. 2009b[2], Yoon et al. 2011[3], Plummer 2015[4]
Chronic Obstructive Pulmonary Disease	2 ICD codes	491.xx, 492.xx, 493.2, 496.xx	J43.x, J44.x	Sensitivity: 70%<br>Specificity: 73%	Gershon et al. 2009a[5], Cooke et al. 2011[6], Yoon et al. 2011[3], Plummer 2015[4]
Interstitial Lung Disease	2 ICD codes, 30 days apart	515, 516.3x, 516.8, 516.9	J84.1x, J84.89, J84.9	PPV: 69.5%<br>Sensitivity: 80.6%<br>Specificity: 98.8%	England et al. 2020[7]
Hypertension	2 ICD codes	401.x, 402.x, 403.x, 404.x, 405.x	I10.x, I11.x, I12.x, I13.x, I14.x	PPV: 81-87%<br>Sensitivity: 73-75%<br>Specificity: 94-95%	Tu et al. 2007[8], Quan et al. 2009[9], Cooke et al. 2011[6], Yoon et al. 2011[3]
Congestive Heart Failure	1 ICD code	398.91, 402.01, 402.11, 402.91, 404.01, 404.03, 404.11, 404.13, 404.91, 404.93, 425.4–425.9x, 428.x	I09.9, I11.0, I13.0, I13.2, I25.5, I42.0, I42.5–I42.9, I43.x, I50.x, P29.0	PPV: 69%<br>Sensitivity: 90%<br>Specificity: 94%	Quan et al. 2009[9], Floyd et al. 2016[10]

© 2024 Savitz DA et al. *JAMA Network Open.*

Myocardial Infarction	1 inpatient ICD code	410.x, 412.x	I21.x, I22.x, I25.2	Sensitivity: 90%<br>Specificity: 99%	Quan et al. 2009[9], Niesner et al. 2013[11], Floyd et al. 2016[10]
Ischemic Stroke	1 inpatient ICD code	433.x, 434.x, 436.x	I63.x	PPV: 80.9%<br>Sensitivity: 52%<br>Specificity: 98%	Niesner et al. 2013[11], Floyd et al. 2016[10]
Hemorrhagic Stroke	1 inpatient ICD code	430.x, 431.x	I60.x, I61.x	PPV: 77.8%<br>Sensitivity: 52%<br>Specificity: 98%	Quan et al. 2009[9], Niesner et al. 2013[11], Floyd et al. 2016[10]

PPV = positive predictive value

Note: Date of diagnosis/onset is the date on which an individual fulfills all criteria of the algorithm (e.g. if the requirement is two ICD codes, the date of diagnosis is the date the second ICD code is recorded).

© 2024 Savitz DA et al. *JAMA Network Open.*

Table S2. Additional cohort sociodemographic and military service characteristics in relation to burn pit exposure

Characteristic	Overall (n=459,381)	No Burn Pit Exposure (n=66,516)	Lowest/Middle Tertile of Burn Pit Exposure (n=262,097)	Highest Tertile of Burn Pit Exposure (n=130,768)
	Number (%)	Number (%)	Number (%)	Number (%)
**Age at VHA enrollment**				
18-24	111084 (24.2)	16012 (24.1)	71598 (27.3)	23474 (18.0)
25-34	197680 (43.0)	24684 (37.1)	108905 (41.6)	64091 (49.0)
35-44	101348 (22.1)	15831 (23.8)	54508 (20.8)	31009 (23.7)
45-54	41513 (9.0)	8331 (12.5)	22485 (8.6)	10697 (8.2)
55+	5203 (1.1)	1307 (2.0)	3068 (1.2)	828 (0.6)
Missing	2553 (0.6)	351 (0.5)	1533 (0.6)	669 (0.5)
**Region of residence**				
Northeast	119912 (26.1)	15398 (23.1)	65319 (24.9)	39195 (30.0)
Midwest	94465 (20.6)	14059 (21.1)	55428 (21.1)	24978 (19.1)
South	131100 (28.5)	20755 (31.2)	73153 (27.9)	37192 (28.4)
West	53592 (11.7)	8038 (12.1)	32989 (12.6)	12565 (9.6)
Missing	60312 (13.1)	8266 (12.4)	35208 (13.4)	16838 (12.9)
**Median household income of Census tract**				
$2500-$46,653	96227 (20.9)	14055 (21.1)	56598 (21.6)	25574 (19.6)
$46,656-$55,067	96197 (20.9)	13348 (20.1)	54037 (20.6)	28812 (22.0)

© 2024 Savitz DA et al. *JAMA Network Open.*

$55,069-$65,699	89447 (19.5)	12756 (19.2)	51397 (19.6)	25294 (19.3)
$65,700-$81,655	82866 (18.0)	12101 (18.2)	46988 (17.9)	23777 (18.2)
$81,658-$250,000	74932 (16.3)	11613 (17.5)	42596 (16.3)	20723 (15.8)
Missing	19712 (4.3)	2643 (4.0)	10481 (4.0)	6588 (5.0)
**Percent with bachelor's degree in Census tract**				
≤17.5	94949 (20.7)	13492 (20.3)	56406 (21.5)	25051 (19.2)
17.6-23.3	93265 (20.3)	12699 (19.1)	52356 (20.0)	28210 (21.6)
23.5-30.4	90274 (19.7)	13237 (19.9)	51057 (19.5)	25980 (19.9)
30.5-41.2	83806 (18.2)	12499 (18.8)	47293 (18.0)	24014 (18.4)
≥41.3	78458 (17.1)	12103 (18.2)	45167 (17.2)	21188 (16.2)
Missing	18629 (4.1)	2486 (3.7)	9818 (3.7)	6325 (4.8)
**VHA Enrollment Priority**				
Group 1-4, 6 (Service connected or disabilities)	339327 (73.9)	49573 (74.5)	192315 (73.4)	97439 (74.5)
Group 5 (low income)	68473 (14.9)	8724 (13.1)	39915 (15.2)	19834 (15.2)
Group 7-8 (low priority, high copays)	51581 (11.2)	8219 (12.4)	29867 (11.4)	13495 (10.3)
**Total deployment duration (days)**				
1-199	46528 (10.1)	15964 (24.0)	30564 (11.7)	0 (0)
200-299	98355 (21.4)	19473 (29.3)	78882 (30.1)	0 (0)
300-399	119286 (26.0)	19524 (29.4)	88475 (33.8)	11287 (8.6)
400-499	57465 (12.5)	5477 (8.2)	23710 (9.0)	28278 (21.6)
500-599	33346 (7.3)	2266 (3.4)	15313 (5.8)	15767 (12.1)

© 2024 Savitz DA et al. *JAMA Network Open.*

600-699	34773 (7.6)	1756 (2.6)	11948 (4.6)	21069 (16.1)
700-799	30234 (6.6)	851 (1.3)	6664 (2.5)	22719 (17.4)
800-899	10714 (2.3)	386 (0.6)	2440 (0.9)	7888 (6.0)
900-999	7992 (1.7)	193 (0.3)	1430 (0.5)	6369 (4.9)
1000+	20688 (4.5)	626 (0.9)	2671 (1.0)	17391 (13.3)
**Time between deployment end and VHA care onset**				
Onset before deployment end	111553 (24.3)	17181 (25.8)	63593 (24.3)	30779 (23.5)
0 years	115348 (25.1)	18408 (27.7)	72547 (27.7)	24393 (18.7)
1 year	64315 (14.0)	7444 (11.2)	35705 (13.6)	21166 (16.2)
2-4 years	106020 (23.1)	13191 (19.8)	56179 (21.4)	36650 (28.0)
5+ years	62145 (13.5)	10292 (15.5)	34073 (13.0)	17780 (13.6)

Abbreviations – VHA: Veterans Health Administration, SD: standard deviation

© 2024 Savitz DA et al. *JAMA Network Open.*

Table S3. Comparison of sociodemographic and military service characteristics between individuals included in the analytic sample and those excluded due to missing data for whom characteristics were available

	**Analytic sample (n=459,381)**	**Excluded due to missing data (n=732,886)**
**Characteristic**	**Number (%)**	**Number (%)**
**Age at VHA enrollment (mean, SD)**		
	31.6 (8.7)	32.3 (9.3)
**Female Sex**		
	57074 (12.4)	80114 (10.9)
**Race/ethnicity**		
White	305996 (66.6)	472095 (64.4)
Black	75258 (16.4)	105654 (14.4)
Other	540 (0.1)	982 (0.1)
Hispanic	52527 (11.4)	87116 (11.9)
Missing	25060 (5.5)	67039 (9.1)
**Region of residence**		
Northeast	119912 (26.1)	208412 (28.4)
Midwest	94465 (20.6)	132244 (18.0)
South	131100 (28.5)	199114 (27.2)
West	53592 (11.7)	93063 (12.7)

© 2024 Savitz DA et al. *JAMA Network Open.*

Missing	60312 (13.1)	100053 (13.7)
**Median household income of Census tract**		
$2500-$46,653	96227 (20.9)	130218 (17.8)
$46,656-$55,067	96197 (20.9)	130311 (17.8)
$55,069-$65,699	89447 (19.5)	136954 (18.7)
$65,700-$81,655	82866 (18.0)	143594 (19.6)
$81,658-$250,000	74932 (16.3)	151516 (20.7)
Missing	19712 (4.3)	40293 (5.5)
**Percent with bachelor's degree in Census tract**		
≤17.5	94949 (20.7)	133551 (18.2)
17.6-23.3	93265 (20.3)	132851 (18.1)
23.5-30.4	90274 (19.7)	137785 (18.8)
30.5-41.2	83806 (18.2)	141248 (19.3)
≥41.3	78458 (17.1)	148872 (20.3)
Missing	18629 (4.1)	38579 (5.3)
**Body Mass Index**		
Underweight/Normal <25	90971 (19.8)	129770 (17.7)
Overweight 25-30	165162 (36.0)	237251 (32.4)
Obese >30	155770 (33.9)	209970 (28.6)
Missing	47478 (10.3)	155895 (21.3)
**Cigarette Smoking**		
Never smoker	186351 (40.6)	272942 (37.2)
Sporadic smoker	93631 (20.4)	134772 (18.4)
Always smoker	105598 (23.0)	118282 (16.1)
Missing	73801 (16.1)	206890 (28.2)

© 2024 Savitz DA et al. *JAMA Network Open.*

**VHA Enrollment Priority**		
Group 1-4, 6 (Service connected or disabilities)	339327 (73.9)	510750 (69.7)
Group 5 (low income)	68473 (14.9)	116001 (15.8)
Group 7-8 (low priority, high copays)	51581 (11.2)	106135 (14.5)
**Calendar year of VHA enrollment**		
Before 2007	69213 (15.1)	181535 (24.8)
2007-2009	107779 (23.5)	132999 (18.1)
2010-2012	134095 (29.2)	139388 (19.0)
After 2012	148294 (32.3)	195663 (26.7)
Missing	69213 (15.1)	83301 (11.4)

Abbreviations – VHA: Veterans Health Administration, SD: standard deviation

© 2024 Savitz DA et al. *JAMA Network Open.*

Table S4. Characteristics of cohort members who received VHA care prior to end of deployment compared to those who only received care after the end of deployment, including breakdown by priority status

Characteristic	Overall (n=459,381)	VHA utilization before deployment end (n=111,647)	VHA utilization after deployment end (n=347,734)
	Number (%)	Number (%)	Number (%)
**Age at VHA enrollment (mean, SD)**			
	31.6 (8.7)	31.2 (8.7)	31.7 (8.8)
**Female sex**			
	57074 (12.4)	13232 (11.9)	43842 (12.6)
**Race/ethnicity**			
White	305996 (66.6)	75333 (67.5)	230663 (66.3)
Black	75258 (16.4)	17044 (15.3)	58214 (16.7)
Other	540 (0.1)	138 (0.1)	402 (0.1)
Hispanic	52527 (11.4)	12237 (11.0)	40290 (11.6)
Missing	25060 (5.5)	6895 (6.2)	18165 (5.2)
**Body Mass Index**			
Underweight/Normal <25	90971 (19.8)	24303 (21.8)	66668 (19.2)
Overweight 25-30	165162 (36.0)	44268 (39.6)	120894 (34.8)
Obese >30	155770 (33.9)	31014 (27.8)	124756 (35.9)
Missing	47478 (10.3)	12062 (10.8)	35416 (10.2)
**Cigarette Smoking**			
Never smoker	186351 (40.6)	48727 (43.6)	137624 (39.6)
Sporadic smoker	93631 (20.4)	23537 (21.1)	70094 (20.2)
Always smoker	105598 (23.0)	21677 (19.4)	83921 (24.1)

© 2024 Savitz DA et al. *JAMA Network Open.*

Missing	73801 (16.1)	17706 (15.9)	56095 (16.1)
**Branch of service**			
Air Force	26316 (5.7)	6030 (5.4)	20286 (5.8)
Army	433065 (94.3)	105617 (94.6)	327448 (94.2)
**Rank**			
Enlisted	408571 (88.9)	96432 (86.4)	312139 (89.8)
Officer/warrant officer	37161 (8.1)	10475 (9.4)	26686 (7.7)
Unknown	13649 (3.0)	4740 (4.2)	8909 (2.6)
**Deployment location**			
Afghanistan only	72448 (15.8)	19938 (17.9)	52510 (15.1)
Iraq only	274810 (59.8)	54378 (48.7)	220432 (63.4)
Both Afghanistan and Iraq	73628 (16.0)	27375 (24.5)	46253 (13.3)
Neither Afghanistan nor Iraq	38495 (8.4)	9956 (8.9)	28539 (8.2)
**Total deployment duration, days, median (IQR)**			
	353 (277-569)	393(280-606)	350(275-543)
**VHA enrollment priority**			
1	94270 (20.5)	10517 (9.4)	83753 (24.1)
2	28606 (6.2)	7289 (6.5)	21317 (6.1)
3	40008 (8.7)	13595 (12.2)	26413 (7.6)
4	510 (0.1)	174 (0.2)	336 (0.1)
5	68473 (14.9)	12786 (11.5)	55687 (16.0)
6	175933 (38.3)	56136 (50.3)	119797 (34.5)
7 or 8	51581 (11.2)	11150 (10.0)	40431 (11.6)
Missing	3293 (0.7)	320 (0.3)	2973 (0.9)

© 2024 Savitz DA et al. *JAMA Network Open.*

**Calendar year of VHA enrollment**			
Before 2007	69213 (15.1)	58975 (52.8)	10238 (2.9)
2007-2009	107779 (23.5)	31386 (28.1)	76393 (22.0)
2010-2012	134095 (29.2)	16271 (14.6)	117824 (33.9)
After 2012	148294 (32.3)	5015 (4.5)	143279 (41.2)

Abbreviations – VHA: Veterans Health Administration, SD: standard deviation, IQR: interquartile range

© 2024 Savitz DA et al. *JAMA Network Open.*

Table S5. Adjusted odds ratios for respiratory and cardiovascular disease by duration of burn pit exposure, 1) excluding those never deployed to Iraq or Afghanistan (n=420,886), 2) excluding those enrolled for VHA care prior to the end of deployment (n=347,734), 3) restricted to those who had an average of more than one healthcare encounter per year of follow-up (n=352,382), and 4) restricted to those with no missing deployment history data (n=389,056).

	Excluding those never deployed to Iraq or Afghanistan (n=420,886)		Excluding those enrolled for VHA care prior to the end of deployment (n=347,734)		Restricted to those who averaged >1 healthcare encounter per year (n=352,382)		Restricted to those with no missing deployment history data (n=389,056)	
	**Adjusted OR***	**95% CI**	**Adjusted OR***	**95% CI**	**Adjusted OR***	**95% CI**	**Adjusted OR***	**95% CI**
**Respiratory Disease**								
**Asthma**								
Overall								
No burn pit exposure	1.00	--	1.00	--	1.00	--	1.00	--
1st Tertile (1-213 days)	1.10	1.03-1.17	1.08	1.03-1.14	1.04	1.00-1.09	1.06	1.02-1.11
2nd Tertile (214-339 days)	1.10	1.04-1.17	1.06	1.01-1.12	1.04	1.00-1.09	1.06	1.02-1.12
3rd Tertile (340-3178 days)	1.15	1.07-1.22	1.06	1.00-1.13	1.06	1.00-1.12	1.13	1.07-1.19

© 2024 Savitz DA et al. *JAMA Network Open.*

aOR per 100 days of exposure	1.013	1.004-1.023	1.002	0.992-1.012	1.009	1.000-1.018	1.016	1.006-1.026
**Chronic Obstructive Pulmonary Disease**								
Overall								
No burn pit exposure	1.00	--	1.00	--	1.00	--	1.00	--
1st Tertile (1-213 days)	1.18	1.03-1.36	1.21	1.08-1.35	1.14	1.03-1.25	1.15	1.04-1.26
2nd Tertile (214-339 days)	1.22	1.06-1.41	1.18	1.05-1.33	1.17	1.06-1.29	1.17	1.05-1.29
3rd Tertile (340-3178 days)	1.28	1.10-1.50	1.17	1.01-1.35	1.19	1.05-1.34	1.17	1.03-1.33
aOR per 100 days of exposure	1.037	1.013-1.061	1.02	0.995-1.047	1.034	1.013-1.056	1.033	1.010-1.057
**Interstitial Lung Disease**								
Overall								
No burn pit exposure	1.00	--	1.00	--	1.00	--	1.00	--
1st Tertile (1-213 days)	1.01	0.69-1.48	1.23	0.88-1.71	1.10	0.83-1.44	1.13	0.85-1.51
2nd Tertile (214-339 days)	1.05	0.71-1.55	1.26	0.89-1.78	1.11	0.83-1.49	1.09	0.81-1.47
3rd Tertile (340-3178 days)	0.84	0.55-1.31	0.88	0.57-1.35	0.86	0.60-1.23	0.82	0.56-1.19

© 2024 Savitz DA et al. *JAMA Network Open.*

aOR per 100 days of exposure	0.995	0.933-1.061	0.998	0.928-1.073	0.996	0.939-1.056	0.995	0.934-1.059
**Cardiovascular Disease**								
**Hypertension**								
Overall								
No burn pit exposure	1.00	--	1.00	--	1.00	--	1.00	--
1st Tertile (1-213 days)	1.07	1.03-1.11	1.02	0.99-1.06	0.99	0.96-1.02	1.01	0.98-1.04
2nd Tertile (214-339 days)	1.12	1.07-1.16	1.04	1.00-1.07	1.04	1.01-1.07	1.06	1.03-1.09
3rd Tertile (340-3178 days)	1.18	1.13-1.23	1.06	1.02-1.11	1.07	1.03-1.11	1.12	1.08-1.16
aOR per 100 days of exposure	1.025	1.019-1.031	1.006	1.000-1.013	1.016	1.011-1.022	1.024	1.018-1.030
**Congestive Heart Failure**								
Overall								
No burn pit exposure	1.00	--	1.00	--	1.00	--	1.00	--
1st Tertile (1-213 days)	0.97	0.85-1.11	1.02	0.91-1.13	0.97	0.88-1.06	0.97	0.88-1.07
2nd Tertile (214-339 days)	0.98	0.86-1.12	0.97	0.87-1.09	0.98	0.89-1.08	0.99	0.90-1.10

© 2024 Savitz DA et al. *JAMA Network Open.*

3rd Tertile (340-3178 days)	0.93	0.80-1.08	0.91	0.79-1.04	0.91	0.81-1.03	0.96	0.84-1.09
aOR per 100 days of exposure	0.992	0.970-1.014	0.975	0.952-0.999	0.990	0.970-1.010	0.998	0.976-1.020
**Myocardial Infarction**								
Overall								
No burn pit exposure	1.00	--	1.00	--	1.00	--	1.00	--
1st Tertile (1-213 days)	1.04	0.79-1.36	1.11	0.89-1.38	1.01	0.84-1.22	1.01	0.83-1.23
2nd Tertile (214-339 days)	0.98	0.74-1.29	0.98	0.77-1.25	0.95	0.78-1.17	1.03	0.84-1.27
3rd Tertile (340-3178 days)	0.96	0.70-1.32	0.85	0.63-1.15	0.90	0.70-1.16	0.99	0.76-1.30
aOR per 100 days of exposure	1.010	0.960-1.063	0.969	0.919-1.023	0.994	0.950-1.041	1.007	0.960-1.056
**Ischemic Stroke**								
Overall								
No burn pit exposure	1.00	--	1.00	--	1.00	--	1.00	--
1st Tertile (1-213 days)	1.04	0.65-1.65	1.53	0.99-2.37	1.27	0.90-1.79	1.39	0.97-2.00
2nd Tertile (214-339 days)	1.15	0.72-1.83	1.88	1.19-2.97	1.38	0.95-1.99	1.56	1.06-2.29

© 2024 Savitz DA et al. *JAMA Network Open.*

3rd Tertile (340-3178 days)	1.19	0.70-2.02	1.84	1.06-3.19	1.34	0.86-2.10	1.74	1.08-2.82
aOR per 100 days of exposure	1.020	0.935-1.114	1.076	0.974-1.189	1.042	0.961-1.130	1.104	1.006-1.211
**Hemorrhagic Stroke**								
Overall								
No burn pit exposure	1.00	--	1.00	--	1.00	--	1.00	--
1st Tertile (1-213 days)	1.22	0.51-2.92	1.00	0.50-2.02	0.96	0.55-1.69	1.01	0.57-1.77
2nd Tertile (214-339 days)	1.38	0.58-3.33	1.25	0.61-2.56	1.03	0.57-1.87	0.93	0.51-1.71
3rd Tertile (340-3178 days)	1.24	0.47-3.26	1.30	0.56-3.05	0.92	0.45-1.89	1.03	0.49-2.17
aOR per 100 days of exposure	0.998	0.876-1.136	0.991	0.859-1.143	0.978	0.869-1.102	1.018	0.880-1.178

Abbreviations – OR: odds ratio, aOR: adjusted odds ratio, CI: confidence interval

*Adjusted for age at enrollment, sex, race/ethnicity, geographic region, median household income and percent with college education of Census tract, obesity status, cigarette smoking, priority for VHA care eligibility, branch of service, year of VHA enrollment, total days of deployment, square of total days of deployment, and cube of total days of deployment

© 2024 Savitz DA et al. *JAMA Network Open.*

Table S6. Odds ratios for respiratory and cardiovascular disease by duration of burn pit exposure, adjusted for all covariates (main analysis), duration of deployment only, or for non-duration covariates only

	Adjusted for all covariates (Table 2 and Table 3)		Adjusted for duration of deployment only		Adjusted for non-duration covariates only	
	Adjusted OR	95% CI	Adjusted OR	95% CI	Adjusted OR*	95% CI
**Respiratory Disease**						
**Asthma**						
Overall						
No burn pit exposure	1.00	--	1.00	--	1.00	--
1st Tertile (1-213 days)	1.05	1.01-1.10	1.02	0.98-1.07	1.05	1.01-1.10
2nd Tertile (214-339 days)	1.05	1.01-1.10	1.04	1.00-1.09	1.04	0.99-1.08
3rd Tertile (340-3178 days)	1.10	1.04-1.16	1.10	1.05-1.16	1.02	0.98-1.07
aOR per 100 days of exposure	1.013	1.004-1.022	1.017	1.008-1.025	0.994	0.988-1.000
**Chronic Obstructive Pulmonary Disease**						
Overall						
No burn pit exposure	1.00	--	1.00	--	1.00	--
1st Tertile (1-213 days)	1.16	1.05-1.27	1.02	0.93-1.12	1.14	1.04-1.26
2nd Tertile (214-339 days)	1.18	1.07-1.30	1.07	0.97-1.18	1.09	0.99-1.20
3rd Tertile (340-3178 days)	1.22	1.08-1.38	1.04	0.92-1.17	1.00	0.91-1.11

© 2024 Savitz DA et al. *JAMA Network Open.*

aOR per 100 days of exposure	1.037	1.016-1.059	1.022	1.002-1.044	0.990	0.976-1.005
**Interstitial Lung Disease**						
Overall						
No burn pit exposure	1.00	--	1.00	--	1.00	--
1st Tertile (1-213 days)	1.11	0.84-1.46	0.96	0.73-1.26	1.10	0.83-1.45
2nd Tertile (214-339 days)	1.11	0.83-1.49	0.96	0.72-1.28	1.08	0.82-1.43
3rd Tertile (340-3178 days)	0.88	0.62-1.26	0.71	0.49-1.01	0.87	0.64-1.18
aOR per 100 days of exposure	0.999	0.941-1.059	0.967	0.911-1.026	0.984	0.943-1.027
**Cardiovascular Disease**						
**Hypertension**						
Overall						
No burn pit exposure	1.00	--	1.00	--	1.00	--
1st Tertile (1-213 days)	1.002	0.97-1.03	0.90	0.88-0.93	0.99	0.97-1.02
2nd Tertile (214-339 days)	1.05	1.02-1.08	0.92	0.90-0.94	1.00	0.97-1.03
3rd Tertile (340-3178 days)	1.10	1.07-1.14	0.96	0.93-0.99	0.94	0.92-0.97
aOR per 100 days of exposure	1.020	1.014-1.025	0.999	0.994-1.004	0.985	0.981-0.988
**Congestive Heart Failure**						
Overall						
No burn pit exposure	1.00	--	1.00	--	1.00	--
1st Tertile (1-213 days)	0.97	0.88-1.07	0.88	0.81-0.97	0.96	0.88-1.05

© 2024 Savitz DA et al. *JAMA Network Open.*

2nd Tertile (214-339 days)	0.99	0.89-1.09	0.90	0.82-1.00	0.92	0.84-1.01
3rd Tertile (340-3178 days)	0.94	0.83-1.06	0.85	0.75-0.95	0.79	0.71-0.87
aOR per 100 days of exposure	0.993	0.974-1.014	0.981	0.962-1.001	0.963	0.949-0.977
**Myocardial Infarction**						
Overall						
No burn pit exposure	1.00	--	1.00	--	1.00	--
1st Tertile (1-213 days)	1.02	0.85-1.23	0.94	0.78-1.13	1.01	0.85-1.21
2nd Tertile (214-339 days)	0.96	0.78-1.17	0.91	0.74-1.11	0.87	0.72-1.06
3rd Tertile (340-3178 days)	0.93	0.72-1.19	0.86	0.67-1.10	0.69	0.56-0.85
aOR per 100 days of exposure	0.998	0.954-1.044	0.994	0.950-1.040	0.935	0.906-0.966
**Ischemic Stroke**						
Overall						
No burn pit exposure	1.00	--	1.00	--	1.00	--
1st Tertile (1-213 days)	1.28	0.91-1.80	1.15	0.82-1.62	1.26	0.89-1.77
2nd Tertile (214-339 days)	1.40	0.97-2.00	1.28	0.89-1.83	1.24	0.88-1.75
3rd Tertile (340-3178 days)	1.44	0.93-2.25	1.29	0.83-2.00	0.92	0.63-1.34
aOR per 100 days of exposure	1.051	0.970-1.139	1.038	0.958-1.126	0.946	0.897-0.997
**Hemorrhagic Stroke**						
Overall						

© 2024 Savitz DA et al. *JAMA Network Open.*

No burn pit exposure	1.00	--	1.00	--	1.00	--
1st Tertile (1-213 days)	0.97	0.55-1.71	0.93	0.53-1.62	0.96	0.55-1.68
2nd Tertile (214-339 days)	1.08	0.60-1.96	1.06	0.59-1.62	0.99	0.56-1.74
3rd Tertile (340-3178 days)	0.97	0.47-1.99	0.95	0.46-1.93	0.82	0.45-1.49
aOR per 100 days of exposure	0.985	0.875-1.109	0.988	0.877-1.112	0.965	0.885-1.053

*Adjusted for age at enrollment, sex, race/ethnicity, geographic region, median household income and percent with college education of Census tract, obesity status, cigarette smoking, priority for VHA care eligibility, branch of service, and year of VHA enrollment

© 2024 Savitz DA et al. *JAMA Network Open.*

Table S7. Sources of covariate data and timing of variable ascertainment

**Variable(s)**	**Source**	**Details**
Birthdate, death data, sex, race, ethnicity	VA Corporate Data Warehouse (CDW)	Age at date of VHA enrollment used for analyses
Inpatient and outpatient encounters	VA CDW	Captured start date of encounter
VA priority status, zip code, region	VA Planning System Support Group	Home zip code at date of VHA enrollment used for analyses
Disease outcomes	VA CDW	ICD-9 and ICD-10 diagnostic codes and associated dates
Body Mass Index	VA CDW	Ascertained at date of VHA enrollment
Smoking	VA CDW	Data from Health Factors survey for each year assigned as "yes" or "no" and aggregated as "always," "never," or "mixed" to designate smoking status.
Branch of service	DoD Defense Manpower Data Center (DMDC)	Only those deployed in Air Force or Army included in analyses
Median income, percent with Bachelor's degree among those age 25+	US Census American Community Survey	Median income and % with Bachelor's degree of home zip code
Total deployment days	DoD DMDC	Squared and cubed total deployment days also included as covariates in regression models

© 2024 Savitz DA et al. *JAMA Network Open.*

Rank	United States Veterans Eligibility Trends and Statistics	Categorized as enlisted, officer, unknown
Cause of death	VA Mortality Data Repository	

eReferences

1. Dombkowski KJ, Wasilevich EA, Lyon-Callo S, Nguyen TQ, Medvesky MG, Lee MA. Asthma surveillance using Medicaid administrative data: a call for a national framework. *J Public Health Manag Pract*. 2009;15(6):485-493. doi:10.1097/PHH.0b013e3181a8c334

2. Gershon AS, Wang C, Guan J, Vasilevska-Ristovska J, Cicutto L, To T. Identifying patients with physician-diagnosed asthma in health administrative databases. *Can Respir J*. 2009;16(6):183-188. doi:10.1155/2009/963098

3. Yoon J, Scott JY, Phibbs CS, Wagner TH. Recent trends in Veterans Affairs chronic condition spending. *Popul Health Manag*. 2011;14(6):293-298. doi:10.1089/pop.2010.0079

4. Plummer AL. International Classification of Diseases, Tenth Revision, Clinical Modification for the Pulmonary, Critical Care, and Sleep Physician. *Chest*. 2015;148(5):1353-1360. doi:10.1378/chest.15-0487

5. Gershon AS, Wang C, Guan J, Vasilevska-Ristovska J, Cicutto L, To T. Identifying individuals with physcian diagnosed COPD in health administrative databases. *COPD*. 2009;6(5):388-394. doi:10.1080/15412550903140865

6. Cooke CR, Joo MJ, Anderson SM, et al. The validity of using ICD-9 codes and pharmacy records to identify patients with chronic obstructive pulmonary disease. *BMC Health Serv Res*. 2011;11:37. doi:10.1186/1472-6963-11-37

7. England BR, Roul P, Mahajan TD, et al. Performance of Administrative Algorithms to Identify Interstitial Lung Disease in Rheumatoid Arthritis. *Arthritis Care Res (Hoboken)*. 2020;72(10):1392-1403. doi:10.1002/acr.24043

8. Tu K, Campbell NR, Chen ZL, Cauch-Dudek KJ, McAlister FA. Accuracy of administrative databases in identifying patients with hypertension. *Open Med*. 2007;1(1):e18-26.

9. Quan H, Khan N, Hemmelgarn BR, et al. Validation of a case definition to define hypertension using administrative data. *Hypertension*. 2009;54(6):1423-1428. doi:10.1161/HYPERTENSIONAHA.109.139279

10. Floyd JS, Blondon M, Moore KP, Boyko EJ, Smith NL. Validation of methods for assessing cardiovascular disease using electronic health data in a cohort of Veterans with diabetes. *Pharmacoepidemiol Drug Saf*. 2016;25(4):467-471. doi:10.1002/pds.3921

© 2024 Savitz DA et al. *JAMA Network Open.*

11. Niesner K, Murff HJ, Griffin MR, et al. Validation of VA administrative data algorithms for identifying cardiovascular disease hospitalization. *Epidemiology*. 2013;24(2):334-335. doi:10.1097/EDE.0b013e3182821e75

© 2024 Savitz DA et al. *JAMA Network Open.*
