# Supplementary material for: Deployment to Military Bases With Open Burn Pits and Respiratory and Cardiovascular Disease
Source: JAMA Netw Open. 2024 Apr 25;7(4):e247629. doi: 10.1001/jamanetworkopen.2024.7629 (PMC11046344; doi:10.1001/jamanetworkopen.2024.7629)
Supplement: Supplement 2. — Data Sharing Statement [file jamanetwopen-e247629-s002.pdf]

Data Sharing Statement

Savitz. Deployment to Military Bases With Open Burn Pits and Respiratory and Cardiovascular Disease. *JAMA Netw Open*. Published April 25, 2024.
doi:10.1001/jamanetworkopen.2024.7629

Data
Data available: No

Additional Information
Explanation for why data not available: The data for this study were provided by the Department of Veterans Affairs and the Department of Defense and those agencies would need to provide data for others to use.
